# A New Method of Haemorrhagic Stroke Detection *Via* Deep Magnetic Induction Tomography

**DOI:** 10.3389/fnins.2021.659095

**Published:** 2021-05-05

**Authors:** Yi Lv, Haijun Luo

**Affiliations:** ^1^College of Electronic and Information Engineering, Shenyang Aerospace University, Shenyang, China; ^2^College of Physics and Electronic Engineering, Chongqing Normal University, Chongqing, China

**Keywords:** magnetic induction tomography (MIT), the forward problem, eddy current, the mass of stroke stimulated (MSS) coils, transcranial magnetic stimulation (TMS)

## Abstract

Hemorrhage imaging is one of the most common applications of magnetic induction tomography (MIT). Depth and the mass of stroke stimulated (MSS) are the most important issues that need to be solved for this application. Transcranial magnetic stimulation (TMS) is a technique belonging to the deep brain stimulation (DBS) field, which aims at overcoming human diseases such as depression. TMS coils, namely, circular, figure-8, and H-coils, play an important role in TMS. Among these, H-coils individually focus on the issues of achieving effective stimulation of deep region. MIT and TMS mechanisms are similar. Herein, for the first time, improved TMS coils, including figure-8 and H-coils, are applied as MIT excitation coils to study the possibility of achieving the mass of stroke stimulated and deep detection through MIT. In addition, the configurations of the detection coils are varied to analyze their influence and determine the optimal coils array. Finally, MIT is used to detect haemorrhagic stroke occurring in humans, and the application of deep MIT to the haemorrhagic stroke problem is computationally explored. Results show that among the various coils, the improved H-coils have MSS and depth characteristics that enable the detection of deep strokes through MIT. Although the detecting depth of the figure-8 coil is weaker, its surface signal is good. The deep MIT technique can be applied to haemorrhagic detection, providing a critical base for deeper research.

## Introduction

Magnetic induction tomography (MIT) is a technique that uses excitation coils to produce an eddy current field in tissues and uses detection coils to detect tissues and image the internal conductivity of the tissue. Owing to its characteristics, MIT is suitable to detect brain strokes. If undetected initially, hemorrhage and ischemic strokes can be fatal or can cause severe non-recoverable damage. Researchers have been simulating MIT application to assess strokes in the human brain, including both two-dimensional (2D) and three-dimensional (3D) models, using various models ranging from a simple sphere model to a real head model. Thus, MIT is advantageous in terms of studying the human brain because existing research is available on it.

In 1993, Al-Zeibak used an electromagnetic system to generate images of simple saline phantoms with electrical conductivities corresponding to fat and fat-free tissue (Al-Zeibak and Saunders, 1993). In 2007, Merwa used MIT to image 2D edema in the human brain (Merwa and Scharfetter, 2007). In 2009, Zolgharni et al. used MIT for the forward modeling and imaging of 3D haemorrhagic cerebral stroke. They used 12 tissues and an operating frequency of 10 MHz, and the conductivity of the stroke tissue was nearly equivalent to that of blood (Zolgharni et al., 2009a,b). Later, Zolgharni et al. used the frequency-difference MIT to compute the same model (Zolgharni et al., 2010). In 2010, Dekdouk et al. investigated the feasibility of detecting a haemorrhagic type stroke using a 3D model *via* simulation. They selected 10 MHz frequency and the stroke conductivity was identical to that of blood (Dekdouk et al., 2010a,b). In 2012, Caeiros et al. used four layers of 3D human head tissue to establish a phantom for simulating a hemorrhage (Caeiros et al., 2012). He used 1 MHz frequency and the conductivity was the same as that of blood, 0.8 S/m. In 2017, Xiao et al. established a 2D human brain model with six layers of tissues to simulate a haemorrhagic stroke and its imaging (Xiao et al., 2017). Therein, the frequencies used were 1 and 10 MHz, and the stroke conductivities were 0.822 and 1.097 S/m, respectively. In 2018, Xiao et al. simulated 2D head models comprising six tissue types with different hemorrhage sizes were simulated, and images of the hemorrhage were reconstructed by multi-frequency difference and single-difference magnetic induction tomography (Zhili et al., 2018). And in 2020, their team proposed a planar MIT method and used it to calculated 3D anatomical head model (Yixuan et al., 2020). In 2020, Ke et al. established one-dimensional quantitative indicators to quantitatively represent intracranial hematoma which can roughly determine the location of the hematoma (Li Ke et al., 2020).

Cerebral stroke is one of the major causes of mortality. There are two types of strokes. An ischaemic stroke, which occurs when a blood vessel becomes occluded, and a haemorrhagic stroke, which occurs when a blood vessel ruptures causing internal bleeding. Conventional imaging techniques for diagnosing strokes such as magnetic resonance imaging (MRI) and computed tomography (CT) are expensive and inaccessible. MIT may be an attractive, low-cost alternative for imaging. The rationale for using MIT is based on the fact that the conductivity of blood is larger than that of other brain tissues, excluding that of cerebrospinal fluid (Zolgharni et al., 2009b). Horesh's study (Horesh et al., 2005) reveals a clear distinction between the three impedances (Ω_*blood*_ < Ω_*brain*_ < Ω_*ischaemia*_), which identify that MIT can distinguish between ischaemic and haemorrhagic strokes (Korjenevsky et al., 2000). The occurrence of cerebral ischemia is usually stopped using thrombotic drugs. However, cerebral hemorrhage is commonly instantaneous, and if not treated in time, it is difficult to heal. Therefore, the use of MIT to compute cerebral hemorrhage has practical significance. The low cost and portability of MIT can significantly assist doctors in diagnosis and help concerned parties better understand stroke conditions.

Transcranial magnetic stimulation (TMS) is a non-invasive brain stimulation technique that is widely used, particularly in clinical neurophysiology, wherein a current pulse is applied to the coil placed around the head to generate an electric field in the human brain via electromagnetic induction to monitor brain activity (Roth et al., 2007; Wagner et al., 2007). The excitation coil is crucial for TMS. Initially, a circular planar coil was proposed for TMS, which was improved to the figure-8 coil to improve detection intensity. However, the figure-8 coil is insensitive to the deep structure of the brain tissue; hence, researchers have designed the H-coil. Although the H-coil is not as strong as the figure-8 coil on the surface, it can detect signals at hippocampus depth (Roth et al., 2002; Zangen et al., 2005). Thus, the figure-8 coil and the H-coil are used in different applications.

Herein, first, a six-layer human head model is established together with three approximate haemorrhagic strokes with different sizes and positions: a larger marginal stroke (LP), a small marginal stroke (SP) and a smaller central stroke (SD). For the head model, a figure-8 coil and a modified H-coil are proposed, and a planar circular coil is applied to the head model. Forty-eight ring detection coils are used to detect signals, and the three types of excitation coils are used to compare the calculation parameters of the head model. We use the detection coil to obtain the MIT signal, comparing the three excitation coils. The proposed improved H-coil is significantly better in depth and the mass of stroke stimulated (MSS) than the other two coils; furthermore, the improved H-coil is significantly different for the MIT signals produced by the ordinary excitation coils for the three strokes. In terms of penetration depth and MSS, the H-coil is best, followed by the figure-8 coil, with the planar circular coil being the worst. The induced electric field strengths of the three different excitation coils are simulated for the three different strokes. Results also show that, of the three coils, the planar circular coil is relatively strong on the surface, and the figure-8 coil is advantageous both in focality and surface signal.

## Materials and Methods

### Frequency Determination

The operating frequency used for the first MIT experiment conducted by Korjenevsky et al. (2000) is 20 MHz, and Scharfetter et al. used 250 kHz frequency for their experimental model (Scharfetter et al., 2001). Afterwards, the researchers proved that the permeability of most biological tissues is close to that of free space (Christ et al., 2010). However, their electrical conductivity is weakly dependent on frequency and can range between 10 kHz and 10 MHz (Mansor et al., 2015).

In fact, many factors influence the operating frequency used in MIT, particularly, in the biomedical field, including displacement current, skin depth (Horesh et al., 2005; Gabriel et al., 2009), and the signal absorption rate (SAR) (Korjenevsky et al., 2000). In addition, the frequency can be considered from another perspective. The purpose of MIT is to obtain the induced signal that is proportional to the amplitude and frequency of excitation. Thus, higher is the frequency, higher is the value that can be gained. High frequency always results in a high signal-to-noise rate (SNR). However, it is not easy to realize with high frequency for the hardware design. And safe is another consideration that limits the frequency. Considering all these factors, for the computational study of cerebral hemorrhage, because the haematoma is higher than the biological tissue, we choose an operating frequency of 10 MHz, which not only meets the requirements of biological tissue safety but can also increase the SNR of MIT (Gencer and Tet, 1999; Voigt et al., 2011). For a given current or voltage, the maximum induced current density must be guaranteed to remain within the biological tissue safety limits in the future experiment study.

### MIT Forward Problem

The forward problem which is considered as solving eddy current model for MIT is to compute the measured signals with the given setup including geometry, distribution of dielectric properties, operating frequency and coil excitation current. Maxwell's equations for MIT with time-harmonic fields and linear materials can be written as follows (Zolgharni et al., 2009b).

(1) $$\begin{array}{l} \nabla \times E=-i\omega \mu H,\;\nabla \times H=J_{s}+E\left(\sigma +i\omega \varepsilon \right),\\ \nabla \cdot \varepsilon E=\rho ,\;\nabla \cdot \mu H=0. \end{array}$$

Here, E and H are the electric and magnetic fields; μ is the permeability; ρ is the electric charge density; and *J*_*s*_ is the current source. The term *iE*ωε corresponds to the displacement current. Applying the temporal gauge *E* = −*iωA*,*B* = ∇ × *A*, the resulting A-formulations of the vector wave equation is given as follows (Soleimani and Lionheart, 2006).

(2) $$\begin{array}{l} \nabla \times \left(\mu^{-1}\nabla \times A\right)+\left(i\omega \sigma -\omega^{2}\varepsilon \right)A=J_{s} \end{array}$$

Once the vector potential is obtained, the induced voltage can be calculated as the line integral of the tangential components of A along a sensor coil

(3) $$\begin{array}{l} V=\underset{Coil}{\oint }E\cdot dl=-i\omega \underset{Coil}{\oint }A\cdot dl \end{array}$$

where d**l** is the length element of the coil.

### Head Model With Hemorrhage

**Figure 1:**
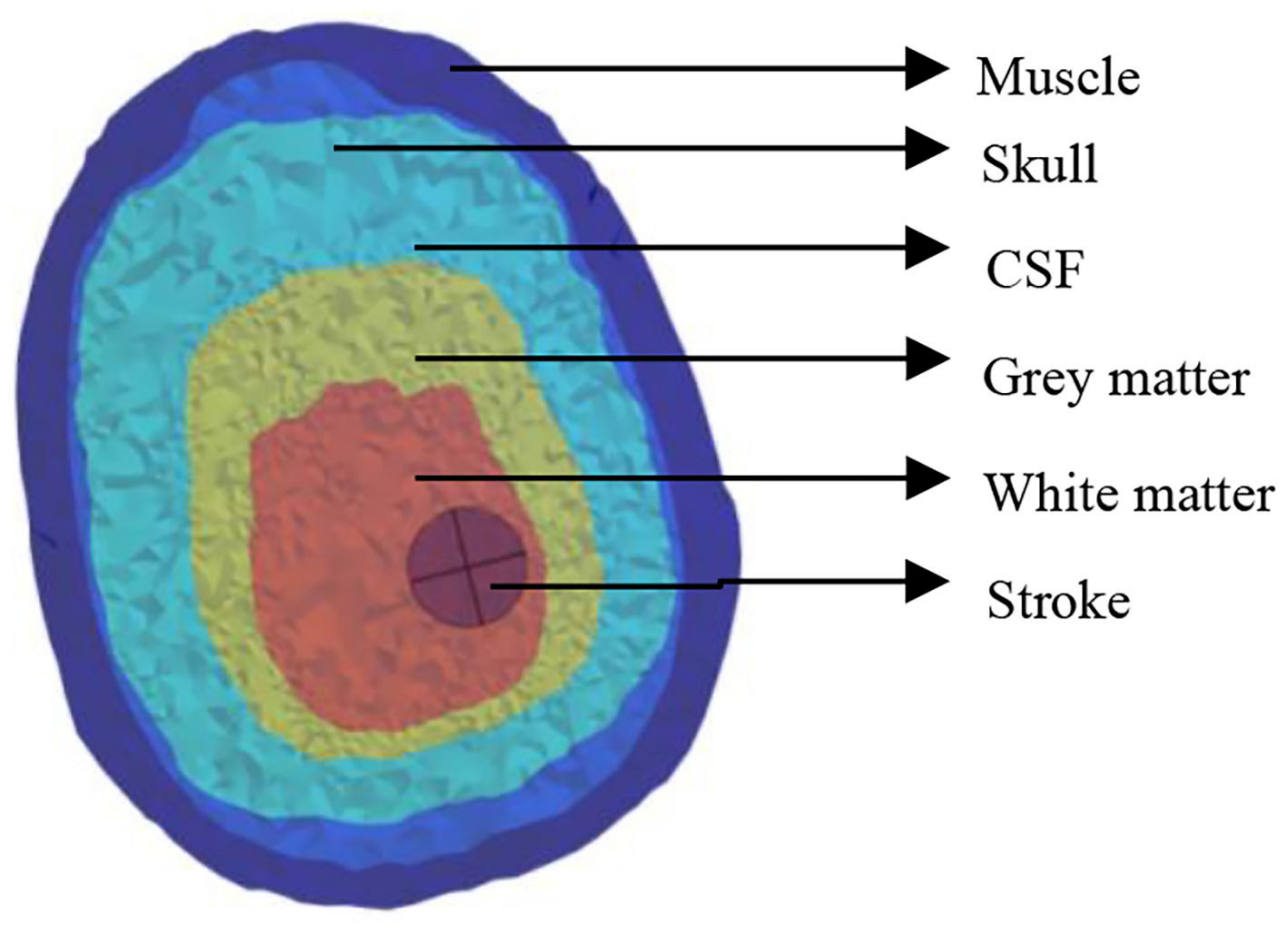
Conductivity of the head's biological tissues.

The electrical properties of the tissue used in the simulations vary with frequency and were obtained from the literature (Table 1) (Lacono et al., 2015). All dielectric properties were assumed to be isotropic, and the relative permeability of all tissues was considered as unity. The human brain structure with cerebral hemorrhage obtained *via* MRI comprises the cerebral cortex, skull, cerebrospinal fluid, gray matter, white matter, and the stroke, as shown in Figure 1. Throughout this study, simulations were conducted at 10 MHz frequency.

**Table 1:** Dielectric properties at 10 MHz assigned to each tissue type in the head model.

**Tissue**	**Conductivity (S/m)**	**Relative permittivity**
Muscle	0.617	171
Skull	0.0829	53.8
CSF	2	109
Gray matter	0.292	320
White matter	0.158	176
Blood	1.10	280
Stroke (75% blood + 25% brain)	0.898	272

For hemorrhage, the stroke was considered to be 75% blood and 25% brain tissue: the conductivity and relative permittivity were calculated as a weighted average of the values for blood and tissue accordingly (e.g., 0.75 × 1.10 + 0.25 + 0.158 = 1.233 S/m for blood in white matter).

**Figure 2:**
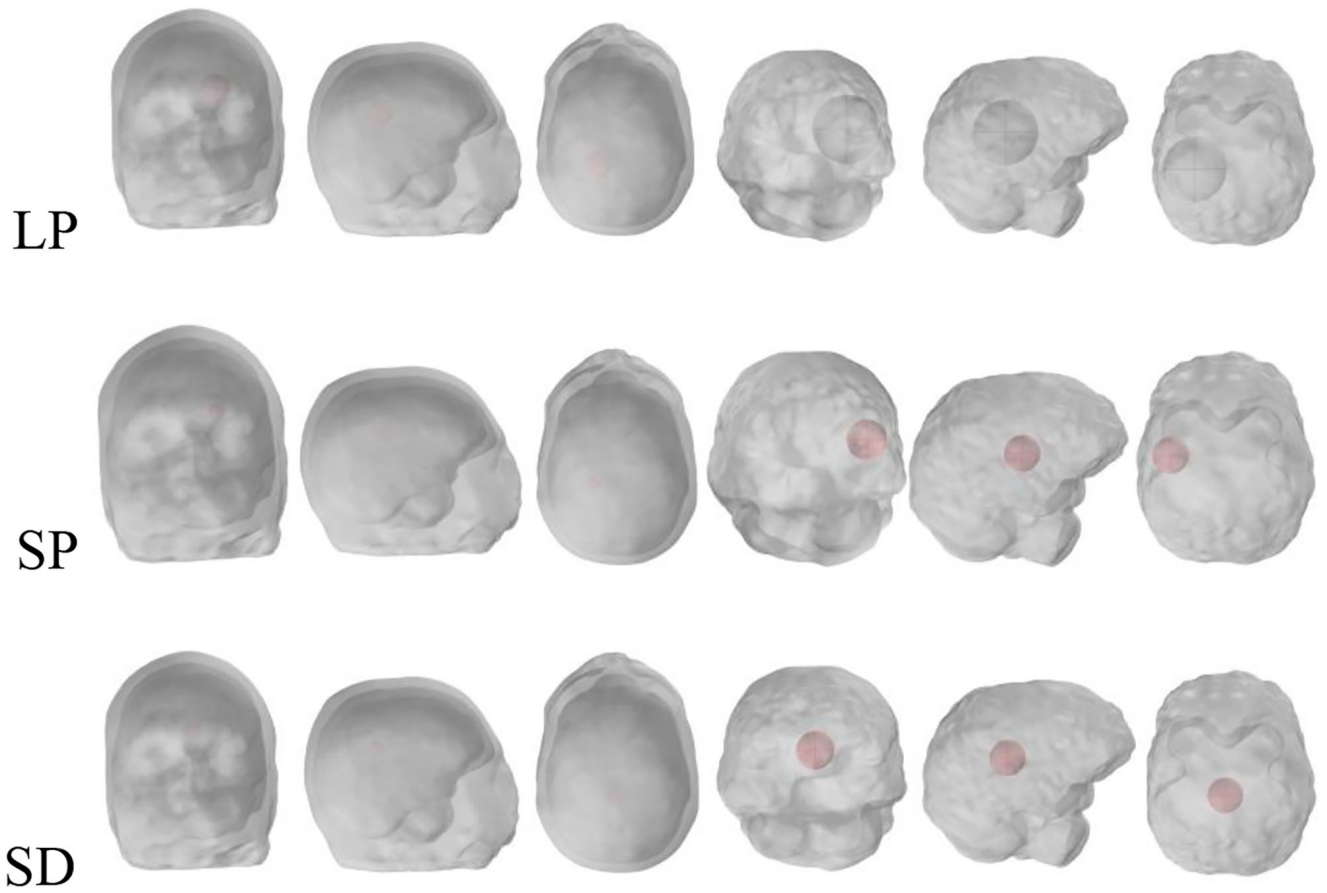
Stroke regions in the left hemisphere of the brain (red), with three different cases: LP, SP and SD.

Simulations were conducted for normal brain condition and six pathological cases, three of which were haemorrhagic—located at the left temporal lobe occupying 4.9, 0.7, and 0.7% of the brain volume. The position of the stroke for hemorrhage can be called a larger marginal stroke (LP), a small marginal stroke (SP) and a smaller central stroke (SD), as shown in Figure 2 (Christ et al., 2010).

### Coils Configurations

#### Excitation Coil

Because the generation mechanism of MIT and TMS is similar, the key coil technology in TMS is introduced into MIT research. The planar circular coil is the technology that was first used in TMS. Then, researchers (Lu and Ueno, 2017) proposed the figure-8 coil because it can be placed on the surface of the biological tissue to produce a large and focal electric field, which is used even today. However, the figure-8 coil is limited to the intracranial and extracranial cerebral cortex, and the electric field generated after excitation of the deep brain tissue is weak. In recent years, neuropsychological disorders have been located in non-surface brain regions; hence, researchers have proposed H-coils that can stimulate deep brain tissue, which is modulated by deep structures rather than cortical modulation for patients. This treatment is more effective than the prior approach.

In this study, these three coil technologies suitable for TMS are applied to MIT cerebral hemorrhage stroke detection. The basis is that the mechanism of the two techniques is similar in the initial stage: injecting current into the coil and applying electromagnetic induction to the biological tissue. Internally induced electromagnetic fields and the MIT midbrain stroke are located deep within the brain. Combining the two techniques enables MIT to clinically detect and monitor cerebral hemorrhage.

**Figure 3:**
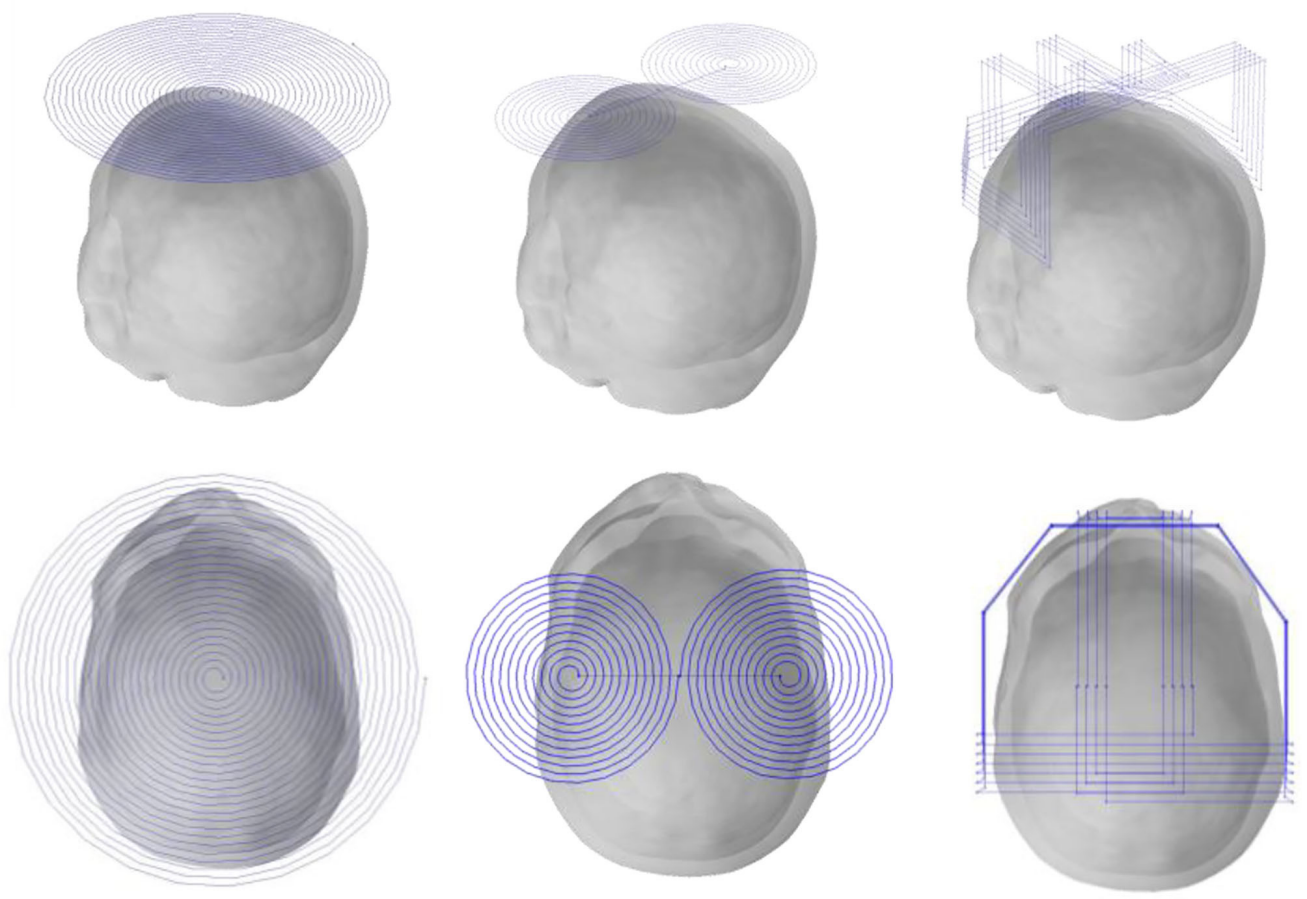
Three different excitation coils.

For the size of the head model, we computed with three coils: the planar circular coil used in the previous MIT, figure-8 coil widely used for TMS, and the recent TMS H-coil, which is an improved version (Figure 3).

The closest distance between the three coils from the top of the head and the edge is ~ 10 mm. The first planar circular coil has 25 turns, the coil center coordinates are (0, −15, and 90 mm), and the maximum diameter is 220 mm; in the figure-8 coil, both coils have 12 turns, the maximum diameter is 106.2 mm, and the center co-ordinates of the two coils are unchanged; finally, in the H-coil, for convenience of design and based on head size, the coil is simply improved according to TMS.

#### Detection Coil

**Figure 4:**
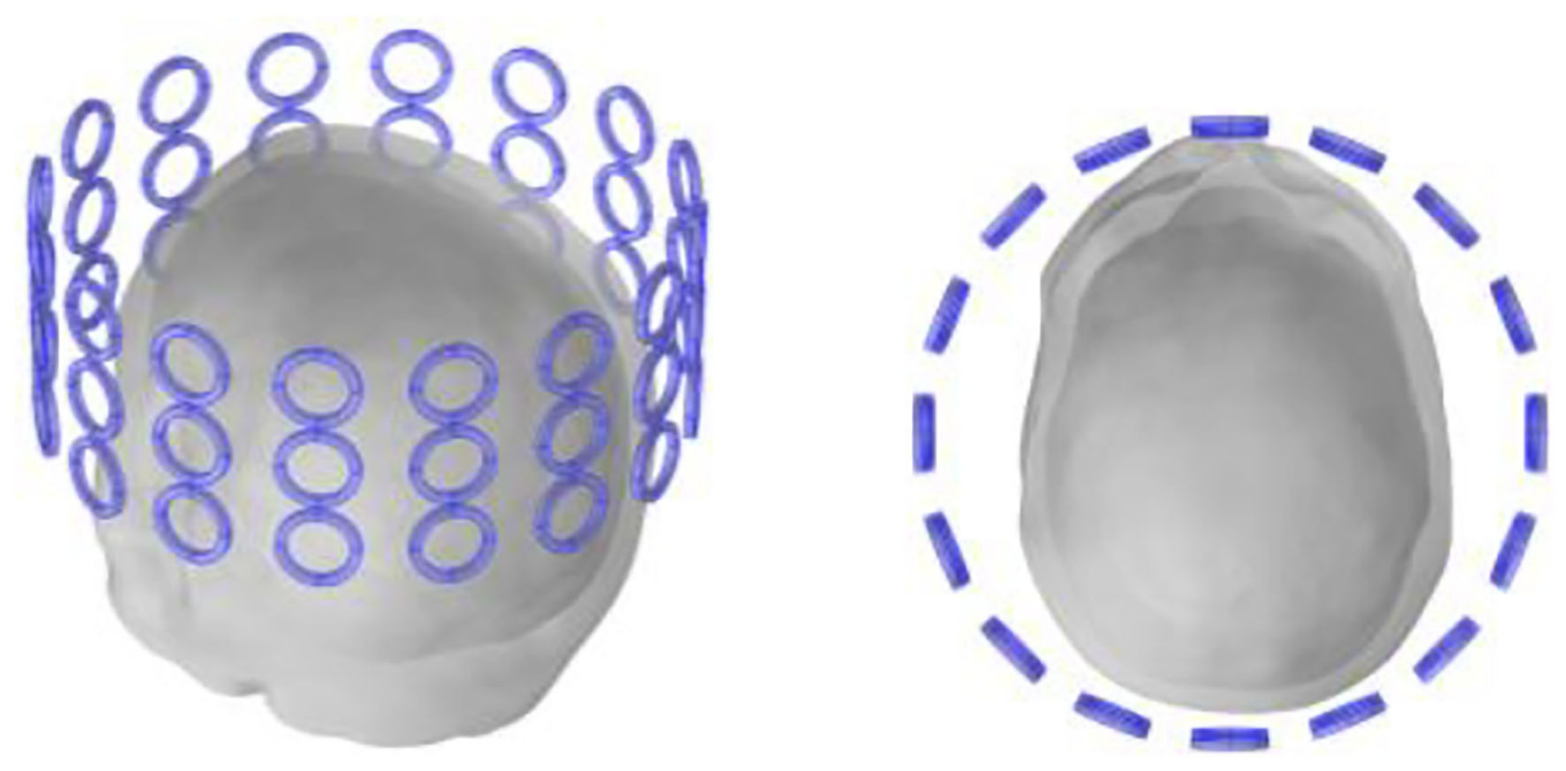
Detection coil.

Previous research (Gürsoy and Scharfetter, 2009; Feldkamp and Quirk, 2017) reveals that the closer the detection coil is to the object to be detected, the larger is the value of the obtained MIT signal; hence, the detection coil of the human head should be placed as close as possible to the human head. In addition, previous studies (Dowrich and Blochet, 2016) show that as the tumor is located in the middle and upper part of the head, the coils in the lower half have little effect on signal detection. All coils use the upper half of the coil, a total of three layers, i.e., 18 small coils per turn. Each coil has a diameter of 28 mm, a width of 4 mm and a coil-to-coil spacing of 2 mm. The material properties of the detection coil are set to copper, as shown in Figure 4.

## Results and Analysis

To accurately calculate the head model containing cerebral edema and facilitate subsequent calculations, the multi-physics software Comsol was used herein. Owing to the complexity of the head model, the requirements for the computer are very fast in terms of speed and accuracy. Hence, we selected a 24G memory Intel i7-4770 computer having a frequency of 3.4 GHz for calculation.

### Penetration Depth and the Mass of Stroke Stimulated (MSS)

The penetration depth and MSS of the excitation coil influences the accuracy of the measurement and the resolution of subsequent imaging. Through comparison, we determine which coil produces a greater penetration depth and MSS.

**Figure 5:**
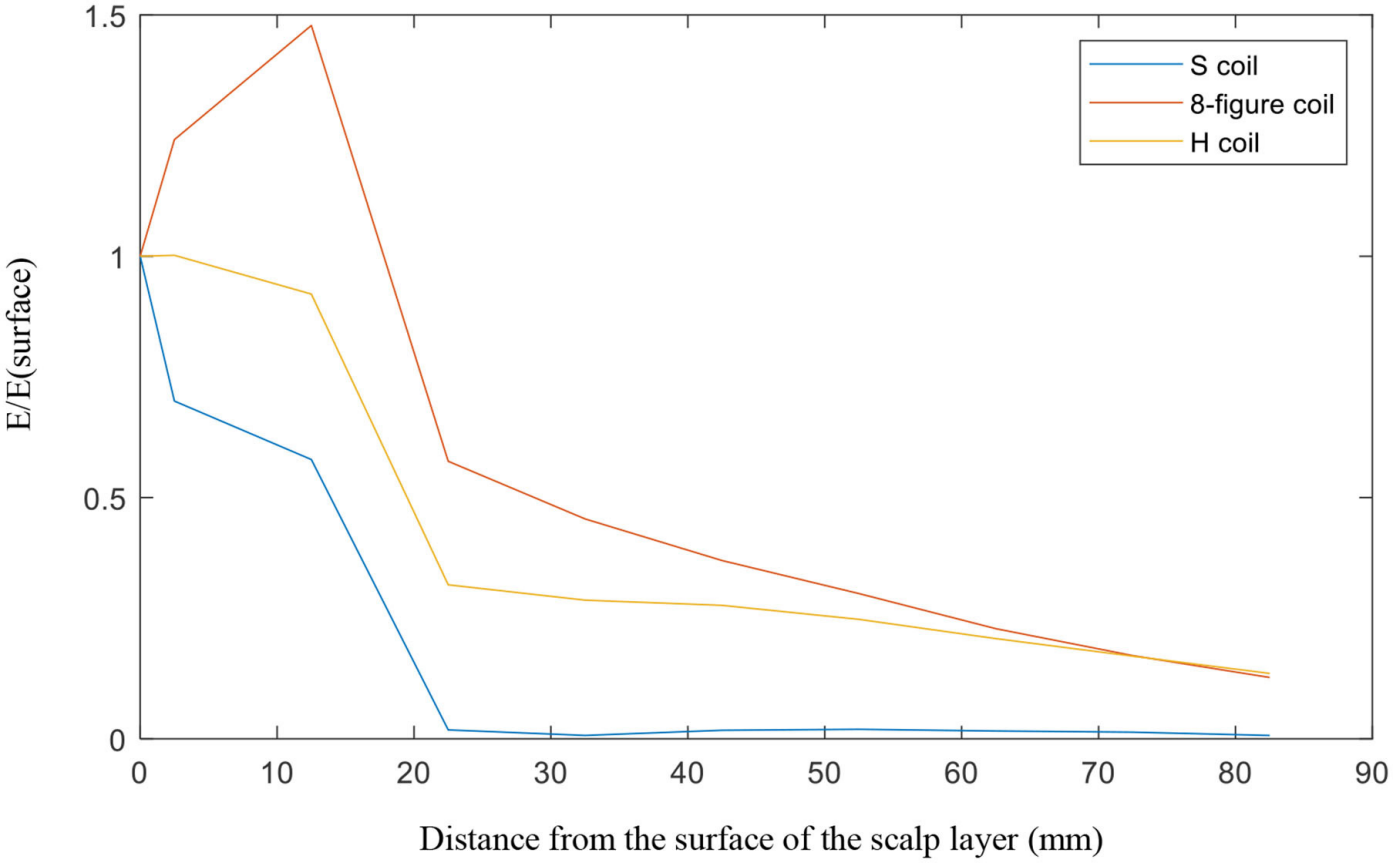
Penetration depth.

For the investigation of penetration depth, SD lumps were examined for the three coils. The distance from the center of the big ball to the top of the head model was 82.5 mm. From the top of the head to the center, we observed the electric field strength generated at the target object. Thus revealed that from the top of the head, the electric field generated by the circular planar coil (S coil) is the largest in the given detection area, up to ~306 V/m, whereas the figure-8 coil's electric field is of only 162 V/m and that of the H-coil is only 58 V/m. However, we found that the planar circular coil which is short for S coil fell extremely fast as it went further. At the center of the head, the electric field strengths of the S, figure-8, and modified H-coils were 23, 21, and 8 V/m, respectively. We divided the electric field by the electric field at the top of the head and then observe their variation. The S coil dropped the fastest, 20 mm from the surface, with the ratio being close to 0; the figure-8 and improved H-coils exhibited a downward trend. However, this trend is relatively good, and the penetration depth of the improved H-coil is relatively better, as shown in Figure 5.

**Figure 6:**
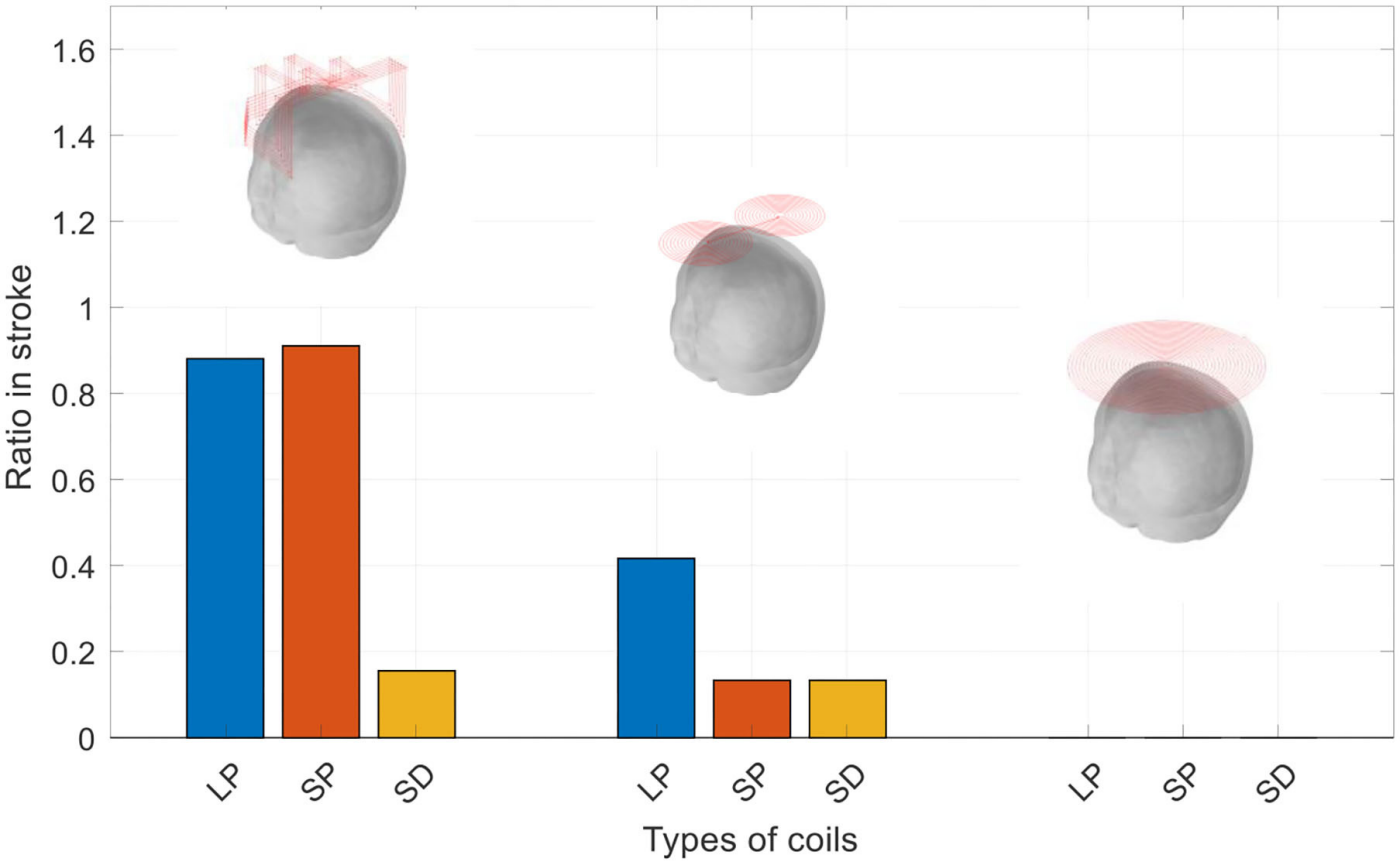
The mass of stroke stimulated.

The mass of stroke stimulated (MSS) was evaluated as the percentage of the electric field strength in the mass as a percentage of the electric field strength of the cortical surface. We examined the three masses of each coil at different percentages. Figure 6 is a graph showing the electric field strength in the three masses of the three coils, which is 40% of the surface of the cortex. As observed in Figure 6, the improved H-coil has the best MSS characteristics, followed by the figure-8 coil, and finally planar circular coil. More than 40% of the electric field strength is zero. To better explore MSS of the coil for MIT, we examined the proportion of the three tumors from 0.4 to 40%. Figure 6 shows that for the improved H-coil, the ratio of 6% of the surface cortical electric field in the mass is 100%; for the figure-8 coil, the ratio of 5% of the surface cortical electric field in the mass is 100%; and for the circular planar coil, the proportion of the cortical field in the mass of 0.5 is 100%. For the starting percentage detected, 40% of the surface cortical electric field begins to have a certain value for the modified H-coil; for the figure-8 coil, 30% begins to have a certain value for the surface cortical electric field; and for the planar circular coil, 2% for the surface electric field starts with a value. In order to better explore the appropriate type of the coil for MIT, we examined the proportions of all three types of strokes ranging from 0.4 to 40%, as shown in Table 2. Therefore, the improved H-coil is the best in terms of both penetration depth and MSS. Consequently, we chose the improved H-coil as the excitation coil for the MIT.

**Table 2:** The mass of stroke stimulated of the three coils.

**Level (%)**	**Improved H coil (%)**			**8-figure coil (%)**			**Spinal coil (%)**		
	**LP**	**SP**	**SD**	**LP**	**SP**	**SD**	**LP**	**SP**	**SD**
40	1.19	0	0	0	0	0	0	0	0
10	33.33	2.22	2.22	32.14	6.67	13.33	0	0	0
6	100	100	100	92.86	100	93.33	0	0	0
1	100	100	100	100	100	100	32.14	4.44	13.33
0.8	100	100	100	100	100	100	47.62	4.44	13.33
0.4	100	100	100	100	100	100	100	100	100

### MIT Signals

**Figure 7:**
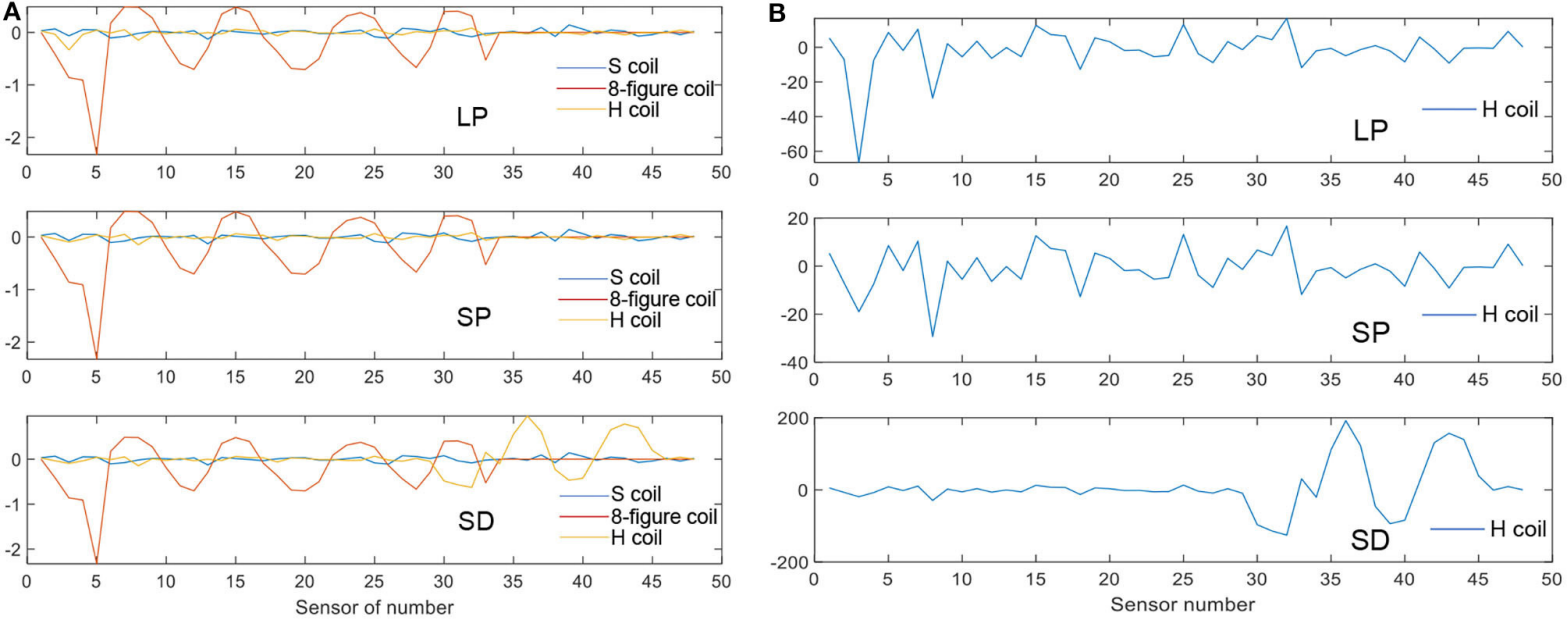
MIT signal. **(A)** MIT Signals of the three coils for three strokes. **(B)** MIT signals of the H-coil for the three strokes.

Simulations were performed for the three coils in the cases of LP, SP, and SD. Here, we have described the H-coil's simulation in the cases of LP, SP, and SD. There are three rows, with each row having 16 coils, providing a total of 48 values according to the number of detection coils. Figure 7A shows that the MIT signals vary with different detection coils for different excitation coils. Figure 7B shows the MIT signals of the improved H-coil under the condition of three strokes. The phase difference, Δϕ, is a measure of the information in the signals due to the hemorrhage:

(4) $$\begin{array}{l} \Delta \phi =\frac{\Delta V}{Vp}=\frac{V2-V1}{Vp} \end{array}$$

Here, *V*_2_ is obtained in the presence of hemorrhage, *V*_1_ in the absence of hemorrhage and *V*_*p*_ is obtained through the direct magnetic coupling between the excitation and sensor coils in the absence of the head.

**Figure 8:**
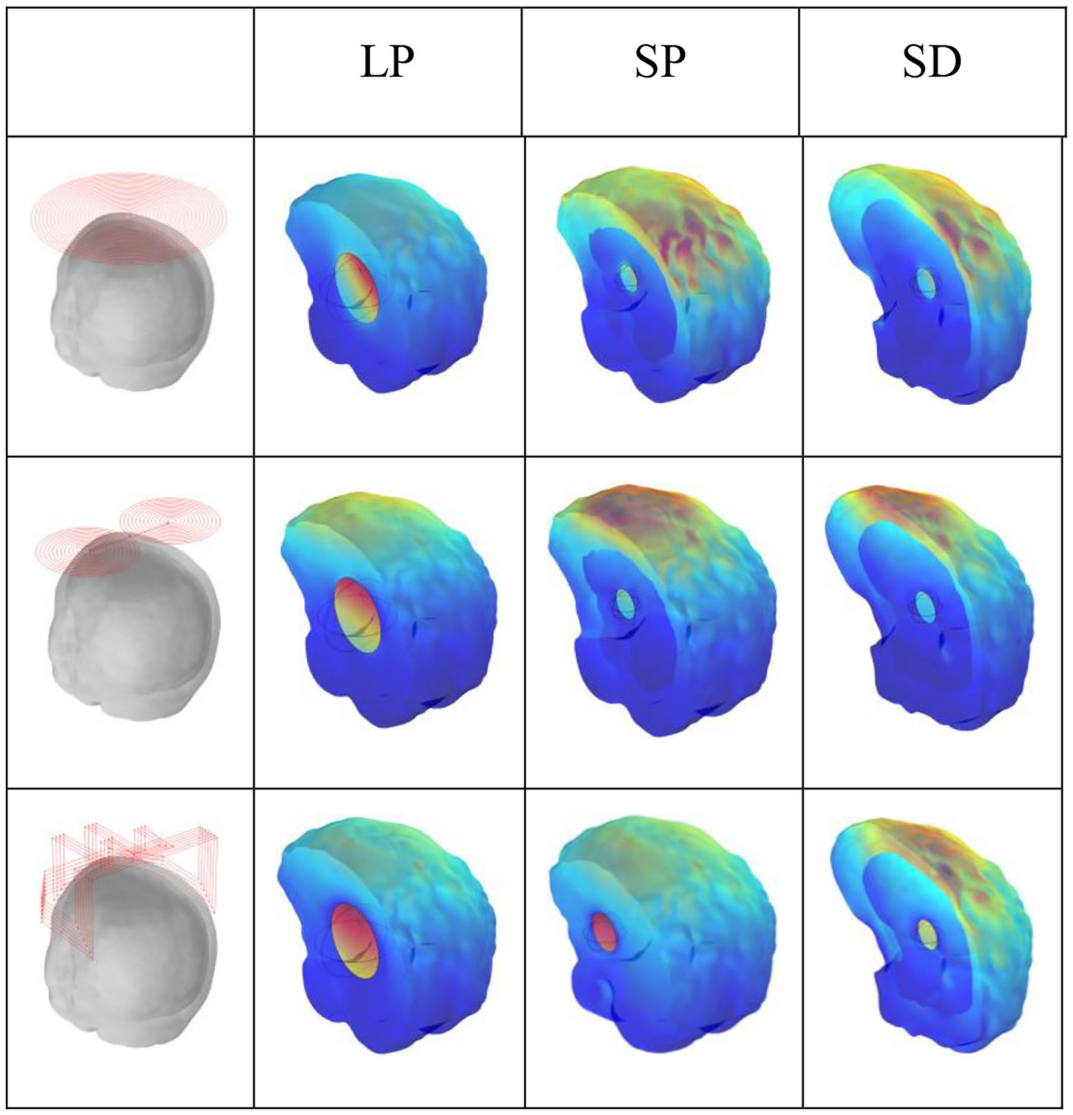
Current density of cerebral haemorrhagic stroke.

The phase value of the figure-8 coil is the largest among the three types of excitation coils. It matches the theory from TMS. The figure-8 coil is the most popular coil used in TMS owing to its high focality. However, this trend differs in the case of SD. The improved H-coil produces great value in the lower detection coils, which are denoted as No. 33 to No. 48. For the SD case, the stroke is set deeper in the brain and is smaller than that of LP, whose results match the theory that H-coils can detect deep signals in the brain compared with other excitation coils. Hence, the improved H-coil can be used in MIT to produce the induced signals that reflect the internal distribution of brain conductivity.

As shown in Figure 7B, the improved H-coil was used to detect changes caused by the LP, SP, and SD in the brain. As the signal was comparatively small, it was enlarged by 200 times. The LP and SP cases are more or less the same except in terms of the value because the position of the stroke is identical although the sizes of the strokes differ. However, for the SD case, although rows one and two are small, there is a drastic increase in row three, even though catch up with LP and SP. Hence, the improved H-coil is suitable for detecting deep signals in the brain through MIT.

MIT Signals of the three coils for three strokes. (2) MIT signals of the H-coil for the three strokes.

### Simulation of Cerebral Hemorrhage Stroke

Figure 8 shows the current densities for fast haematoma, white matter, and gray matter in the condition of the three excitation coils. For TMS technology, the figure-8 coil produced the largest electric field on the cortical surface, whereas the H-coil produced a smaller electric field; however, the H-coil exhibited good penetration depth and can measure brain depth tissue, which is lacking in the figure-8 coil. Let us examine the current density generated by the three different excitation coils in brain tissue during MIT to reflect whether the characteristics of the three coils are consistent. Figure 8 shows that for the penetration depth, in the case of haematoma in three different positions and sizes, the improved H-coil produces the highest current density in the mass, and the current density generated by the planar circular coil is the smallest, thereby showing improvement. The penetration depth of the H-coil is the best performance; for the signal generated on the cortical surface, the figure-8 coil produces the highest current density on the surface of the brain tissue, and the improved H-coil produces the current on the surface of the brain tissue. This is in complete agreement with the improved H-coil and the characteristics of the figure-8 coil in TMS technology.

## Discussion

Magnetic induction imaging was proposed in 1993 for its low cost, contactless, non-invasive, and portable properties. The original purpose of this technology was to overcome the shortcomings of EIT technology to achieve absolute conductivity, real-time performance, skull penetration and low contrast (Holder, 1992). The biggest problem with MIT technology is the low spatial resolution, which is determined by the MIT generation principle. Therefore, extracting the weaker eddy current signals from the stronger main signal and increasing the sensitivity are urgent issues that need to be solved using MIT. In addition, the ultimate goal of MIT is application to medical treatment. Therefore, improving hardware to meet medical needs is also a research concern (Ma and Soleimani, 2017). In recent years, scholars have focused on the improvement of coil sensors.

Because the size and shape of the excitation coils influences the sensitivity of the field which could be induced in the target objects, and the location of the detection coils affects the quality of the image reconstruction, implying that research on coil sensors may be the key to MIT. By improving the sensor, possibly, the previous eddy current signal, low contrast, spatial resolution, and eddy current signal extraction may be improved or solved.

TMS is a non-invasive brain active technology widely used in clinical neurophysiology; in particular, deep TMS technology is widely used in clinical and therapeutic approaches. However, scholars are still exploring the depth of TMS technology in induction electric field distribution, wherein the key to this technology are the planar circular, figure-8, and H-coils used with deep TMS. The mechanism of MIT and TMS signal generation is similar. Notably, the TMS coil that has been applied in clinical approaches has been applied to MIT for the first time. The two technologies are combined to detect and image brain tissue.

From the perspective of MIT, considering the actual cerebral hemorrhage problem, MIT has the technical basis to solve cerebral hemorrhage. At simulation level, MIT has attracted the attention of scholars. If this problem can be solved through MIT, it can definitely be used for clinical testing and self-monitoring of patients, which is a great contribution. The application of the coil in deep TMS to MIT will direct our future work.

Herein, at 10 MHz, including six layers of biological tissue of the haematoma and using three coils applied to TMS, MIT was applied to detect different haematomas. The simulation results show that H-coil has obvious advantages in depth measurement and MSS for deep tissue detection. The results obtained by the scholars in TMS technology are similar. In future works, these three coils can be imaged using MIT imaging algorithms to quantify their contribution to spatial resolution. In addition, as TMS coil technology can be applied to MIT, we use deep TMS technology as the basis. Extending the research on the depth excitation coil in addition to the depth of penetration and MSS, the MIT imaging algorithm can also be used to image the haematoma and perform comprehensive quantitative comparisons of the systems; furthermore, we need to apply MIT to the 3D head print model. Preliminary laboratory specific tests must be conducted on animals; then, beginning with TMS technology, which is a popular neuromodulation technology at this stage, we can investigate whether MIT technology can detect changes in nerve stimulation and then perform imaging. These are the directions for our future research.

## Conclusion

At an operating frequency of 10 MHz, for cerebral hemorrhage, three types of haematoma with varying sizes and positions were calculated using three coils suitable for TMS technology, aiming to solve the detection and focusing of deep brain tissue. The results show that the H-coil has the best penetration and MSS performance, and can detect the internal tissue signal of the brain. Although the figure-8 coil has poor penetration depth, the signal amplitude and the focality of the surface layer are advantageous. This deep MIT technology can be used for cerebral hemorrhage detection, providing a feasible basis for future research, and for combining the two technologies of MIT and TMS to provide a deeper direction.

## Data Availability Statement

The raw data supporting the conclusions of this article will be made available by the authors, without undue reservation.

## Author Contributions

YL has proposed the conception, established the model and did the simulations computational experiments, and analyzed the results. HL has discussed and confirmed the conception, proposed some methods, and algorithm to solve some problems. He also discussed and modified the paper details. All authors contributed to the article and approved the submitted version.

## Conflict of Interest

The authors declare that the research was conducted in the absence of any commercial or financial relationships that could be construed as a potential conflict of interest.

## References

[B1] Al-Zeibak S. Saunders N. H. (1993). A feasibility study of *in vivo* electromagnetic imaging. Phys. Med. Biol. 38, 151–160. 10.1088/0031-9155/38/1/011 8426866

[B2] Caeiros J. Martins R. C. Gil B. (2012). “A new image reconstruction algorithm for real-time monitoring of conductivity and permeability changes in magnetic induction tomography,” in 2012 Annual International Conference of the IEEE Engineering in Medicine and Biology Society (San Diego), 6239–6242. 10.1109/EMBC.2012.6347420 23367355

[B3] Christ A. Kainz W. Hahn E. G. Chen J. (2010). The virtual family—development of surface-based anatomical models of two adults and two children for dosimetric simulations, Phys. Med. Biol. 55, N23–N38. 10.1088/0031-9155/55/2/N01 20019402

[B4] Dekdouk B. Ktistis C. Armitage D. W. Peyton A. J. (2010a). Assessing the feasibility of detecting a haemorrhagic type stroke using a 16 channel magnetic induction system. J. Phys. Conf. Ser. 224, 1–4. 10.1088/1742-6596/224/1/012047

[B5] Dekdouk B. Yin W. L. Ktistis C. Peyton A. J. (2010b). A method to solve the forward problem in magnetic induction tomography based on the weakly coupled field approximation. IEEE Trans. Bio-Med. Eng. 57, 914–921. 10.1109/TBME.2009.2036733 19932988

[B6] Dowrich T. C. Blochet Holder D. (2016). *In vivo* bioimpedance changes during haemorrhagic and ischaemic stroke in rats: towards 3D stroke imaging using electrical impedance tomography. Physiol. Meas. 37, 765–784. 10.1088/0967-3334/37/6/765 27200510

[B7] Feldkamp J. R. Quirk S. (2017). Coil geometry effects on scanning single coil magnetic induction tomography. Phys. Med. Biol. 62, 7097–7113. 10.1088/1361-6560/aa807b 28718776

[B8] Gabriel C. Peyman A. Grant E. H. (2009). Electrical conductivity of tissue at frequency below 1MHz. Phys. Med. Biol. 54, 4863–4878. 10.1088/0031-9155/54/16/002 19636081

[B9] Gencer N. G. Tet M. N. (1999). Electrical conductivity imaging *via* contactless measurements. IEEE Trans. Med. Imag. 187:79046. 10.1109/42.790461 10504095

[B10] Gürsoy D. Scharfetter H. (2009). Optimum receiver array design for magnetic induction tomography. IEEE Trans Biomed Eng. 56, 1435–1441. 10.1109/TBME.2009.2013936 19203883

[B11] Holder D. S. (1992). Electrical impedance tomography of brain function. Brain Topogr. 5, 87–93. 10.1007/BF01129035 1489654

[B12] Horesh L. Gilad O. Romsauerova A. Holder D. S. (2005). “Stroke type differentiation by multi-frequency electrical impedance tomography —a feasibility study,” in Proceedings of 3rd European Medical and Biological Engineering Conference (Prague), 1–4.

[B13] Korjenevsky A. Cherepenin V. Sapetsky S. (2000). Magnetic induction tomography: experimental realization. Physiol. Meas. 21, 365–368. 10.1088/0967-3334/21/1/311 10720003

[B14] Lacono M. I. Neufeld E. Akinnagbe E. Bower K. Wolf J. Oikonomidis I. V.. (2015). MIDA: A multimodal imaging-based detailed anatomical model of the human head and neck. PLoS ONE 10. 10.1371/journal.pone.0124126 25901747 PMC4406723

[B15] Li Ke W. Zu Q. D. Jia C. Xiaodi D. (2020). A bio-impedance quantitative method based on magnetic induction tomography for intracranial hematoma. Med. Biol. Eng. Comput. 58, 857–869. 10.1007/s11517-019-02114-7 32060798

[B16] Lu M. Ueno A. (2017). Comparison of the induced fields using different coil configurations during deep transcranial magnetic stimulation. PLoS ONE 12, 1–12. 10.1371/journal.pone.0178422 28586349 PMC5460812

[B17] Ma L. Soleimani M. (2017). Magnetic induction tomography methods and applications: a review. Meas. Sci. Technol. 28, 1–12. 10.1088/1361-6501/aa7107

[B18] Mansor M. S. B. Zakaria Z. Balkhis I. Rahim R. A. (2015). Magnetic induction tomography: a brief review. J. Teknol. 73, 91–95. 10.11113/jt.v73.4252

[B19] Merwa R. Scharfetter H. (2007). “Magnetic induction tomography: a feasibility study of brain oedema detection using a finite element human head model,” in 13th International Conference on Electrical Bioimpedance and the 8th Conference on Electrical Impedance Tomography, Vol., 17. (Graz)384–387.

[B20] Roth Y. Padberg F. Zangen A. (2007). Transcranial magnetic stimulation of deep brain region: principles and methods. Recent Adv. Biol. Psychiatr. 23, 204–224. 10.1159/000101039

[B21] Roth Y. Zangen A. Hallett M. (2002). A coil design for transcranial magnetic stimulation of deep brain regions. J. Clin. Neurophysiol. 19, 361–370. 10.1097/00004691-200208000-00008 12436090

[B22] Scharfetter H. Lackner H. K. Rosell J. (2001). Magnetic induction tomography: hardware for multi-frequency measurements in biological tissues. Physiol. Meas. 22, 131–146. 10.1088/0967-3334/22/1/317 11236874

[B23] Soleimani M. Lionheart W. R. B. (2006). Absolute conductivity reconstruction in magnetic induction tomography using a nonlinear method. IEEE Trans. Med. Imag. 25, 1521–1530. 10.1109/TMI.2006.884196 17167989

[B24] Voigt T. Katscher U. Doessel O. (2011). Quantitative conductivity and permittivity imaging of the human brain using electric properties tomography. Magn. Resonan. Med. 66, 456–466. 10.1002/mrm.22832 21773985

[B25] Wagner T. Valero-Cabre A. Pascual-Leone A. (2007). Non-invasive human brain stimulation. Ann. Rev. Biomed. Eng. 9, 527–565. 10.1146/annurev.bioeng.9.061206.133100 17444810

[B26] Xiao Z. L. Tan C. Dong F. (2017). Effect of inter-tissue inductive coupling on multi-frequency imaging of intracranial hemorrhage by magnetic induction tomography. Meas. Sci. Technol. 28:aa7504. 10.1088/1361-6501/aa7504

[B27] Yixuan C. Chao T. Feng D. (2020). Combined planar magnetic induction tomography for local detection of intracranial hemorrhage. IEEE Trans. Instrum. Meas. 70:3011621. 10.1109/TIM.2020.3011621

[B28] Zangen A. Roth Y. Voller B. Hallett M. (2005). Transcranial magnetic stimulation of deep brain regions: evidence for efficacy of the H-coil. Clin. Neurophysiol. 116, 775–779. 10.1016/j.clinph.2004.11.008 15792886

[B29] Zhili X. Chao T. Feng D. (2018). Multi-frequency difference method for intracranial hemorrhage detection by magnetic induction. Physiol. Meas. 39:aac09c. 10.1088/1361-6579/aac09c 29701181

[B30] Zolgharni M. Griffiths H. Ledger P. D. (2010). Frequency-difference MIT imaging of cerebral haemorrhage with a hemispherical coil array: numerical modelling. Physiol. Measu. 31, 111–114. 10.1088/0967-3334/31/8/S09 20647622

[B31] Zolgharni M. Ledger P. D. Armitage D. W. Holder D. Griffiths H. (2009a). Imaging cerebral haemorrhage with magnetic, induction tomography: numerical modeling. Physiol. Meas. 30, 187–200. 10.1088/0967-3334/30/6/S13 19491437

[B32] Zolgharni M. Ledger P. D. Griffiths H. (2009b). Forward modelling of magnetic induction tomography: a sensitivity study for detecting haemorrhagic cerebral stroke. Med. Biol. Eng. Comput. 47, 1301–1304. 10.1007/s11517-009-0541-1 19834756

